# Subclinical Infections with Crimean-Congo Hemorrhagic Fever Virus, Turkey

**DOI:** 10.3201/eid1804.111374

**Published:** 2012-04

**Authors:** Hürrem Bodur, Esragül Akinci, Sibel Ascioglu, Pinar Öngürü, Yavuz Uyar

**Affiliations:** Ankara Numune Education and Research Hospital, Ankara, Turkey (H. Bodur, E. Akinci, P. Öngürü);; Hacettepe University Medical School, Ankara (S. Ascioglu);; Refik Saydam National Public Health Agency, Ankara (Y. Uyar)

**Keywords:** Crimean-Congo hemorrhagic fever, Crimean-Congo hemorrhagic fever virus, viruses, seroprevalence, subclinical infections, Turkey

## Abstract

To investigate Crimean-Congo hemorrhagic fever virus in Turkey, we conducted a seroepidemiologic survey during January–April 2009. Seroprevalence of infection was 10% in a sample from an outbreak region and increased with patient age, indicating that the virus had been previously present in Turkey. We also estimated that 88% of infections were subclinical.

Crimean-Congo hemorrhagic fever virus (CCHFV) infection was first recognized in Turkey in 2002. Since that time, the number of diagnosed cases has increased to the magnitude of an outbreak with major public health consequences (*1**,**2*). Although the clinical spectrum may change from asymptomatic infection to severe hemorrhagic disease, most studies to date have included only symptomatic cases (*1**–**6*). Therefore, the true incidence of infection with the virus, the full spectrum of severity of disease, and its epidemiologic features are unknown. The purpose of our study was to investigate the seroprevalence of CCHFV infection in a sufficiently large sample representative of the region affected during this outbreak in Turkey and to describe the main epidemiologic features, including the proportion of subclinical cases.

## The Study

**Figure 1:**
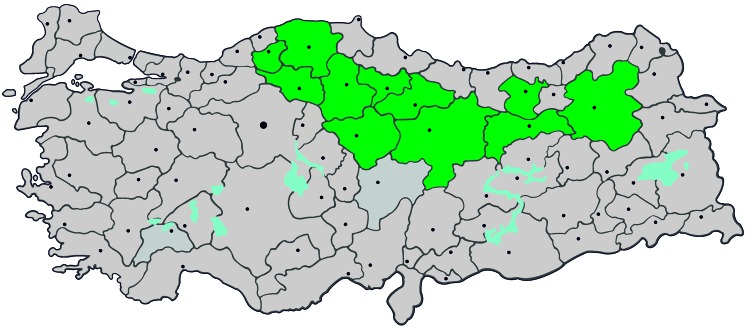
Provinces in Turkey where study was conducted and to which Crimean-Congo hemorrhagic fever virus is endemic (green), January–April 2009. Gray indicates other provinces and dots indicate major cities.

During January–April 2009, we conducted a survey that included obtaining venous blood samples from participants. The study sample was selected by using a random geographic cluster sampling stratified by age and sex from rural residential areas of Turkey where 99.5% of CCHF cases were reported (Figure 1). Only adults >18 years of age who were living in the study area for >1 year were eligible for the study. The study was approved by the Central Ethics Committee, and informed consent was obtained from all participants. A study questionnaire included questions on demographics, socioeconomic status, behavior characteristics, medical history, known risk factors for CCHFV infection, and participants’ awareness of the outbreak and infection prevention methods.

Serum samples were tested for IgG against CCHFV at the Virology Reference Laboratory of the Refik Saydam National Public Health Agency, (Ankara, Turkey) by using a commercial ELISA kit (Vector-Best, Novosibrisk, Russia). Although the sensitivity and specificity of the kit were not specified by the manufacturer, studies that used this method have reported a sensitivity of 87%–98.3% and a specificity of 99%–100% (*7**,**8*). We defined subclincial cases as those in persons who were seropositive although they were not given a diagnosis or had not had severe symptoms compatible with CCHF at any time.

In addition, we compared information in our database with that in the database of reported cases at the Ministry of Health, Turkey. We used the χ^2^ test, *t* test, and Mann-Whitney U test for univariate statistical comparisons, as appropriate. Multivariable logistic regression was used to assess independent risk factors for seropositivity. We used the direct standardization method to adjust age-specific seropositivity rates for our study population with the age composition of the entire outbreak region. This adjustment enabled us to calculate expected numbers of infected (seropositive) persons in the outbreak region (Technical Appendix). Clinically diagnosed cases were compared with expected numbers of infections, and an observed:expected ratio was calculated.

The survey included 3,671 adults of whom 3,557 (97%) provided blood samples for serologic analysis. The mean ± SD age of the study population was 44.3 ± 16.2 years, the female:male ratio was 1.04 (51%:49%), and the most common occupation was farming (52.4%). Only 18.2% had a history of tick bite.

**Figure 2:**
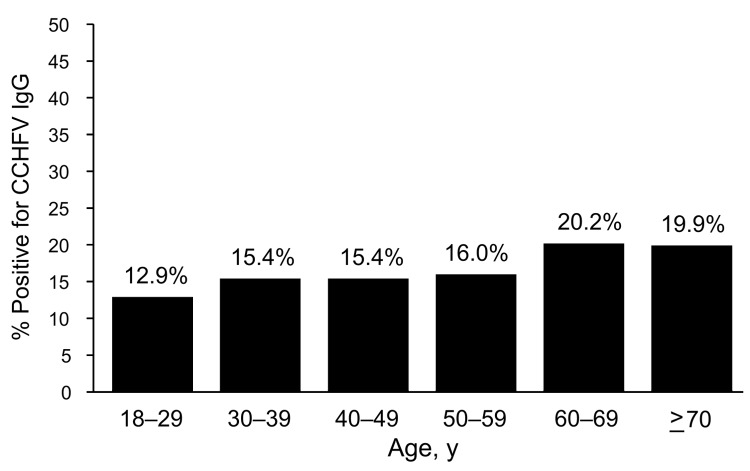
Distribution of Crimean-Congo hemorrhagic fever virus (CCHFV)–positive persons, by age group, Turkey, January–April 2009. p<0.001, by trend test.

Of 3,557 serum samples tested, 356 (10%) were positive for IgG against CCHFV. Mean ± SD age was 43.4 ± 16.2 years for seronegative persons and 52 ± 17.1 years for seropositive persons (p<0.001). Categorizing persons by age in 10-year intervals showed that seropositivity increased with age (p<0.001) (Figure 2). Univariate analysis showed that seropositive persons had less education (p<0.001), were more likely to be involved in farming, (p<0.001), and had a higher frequency of tick bites (p<0.001) than seronegative persons (Table 1). Animal husbandry as an occupation and a history of hunting were not more frequent among seropositive persons than among seronegative persons. A high proportion of seropositive persons (73.8%) and seronegative persons (71.3%), claimed that they had sufficient information about the infection and how to protect themselves (p = 0.329). Multivariable analysis results showed that an age >60 years, less schooling, farming as an occupation, and a history of tick bites were independent risk factors for seropositivity (Table 2).

**Table 1:** Characteristics of study population tested for CCHFV, Turkey, January–April 2009*

Characteristic	Seronegative, n = 3,201	Seropositive, n = 356	p value
Sex			
F	1,634 (51.0)	168 (47.2)	0.167
M	1,567 (49.0)	188 (52.8)	
Age, y, mean ± SD	43.38 ± 15.81	51.97 ± 17.14	<0.001
18–29	765 (23.9)	46 (12.9)	<0.001
30–39	721 (22.5)	55 (15.4)	
40–49	616 (19.2)	55 (15.4)	
50–59	511 (16.0)	57 (16.0)	
60–69	352 (11.0)	72 (20.2)	
≥70	236 (7.4)	71 (19.9)	
Education			
None	607 (19.0)	105 (29.7)	<0.001
Elementary school	1,873 (58.8)	214 (60.6)	
High school or university	707 (22.2)	34 (9.6)	
Occupation			
Unemployed	618 (19.5)	66 (18.9)	<0.001
Farming	1,186 (37.5)	174 (49.9)	
Animal husbandry	553 (17.5)	58 (16.6)	
Other†	809 (25.5)	51 (14.6)	
Persons living in same residence, mean ± SD	5.01 ± 2.54	5.28 ± 2.97	0.177
History of hunting	548 (17.6)	54 (15.5)	0.325
History of tick bite	540 (17.0)	105 (29.7)	<0.001
Sufficiently informed about CCHFV	2,269 (71.3)	261 (73.7)	0.329

*Values are no. (%) unless otherwise indicated. CCHFV, Crimean-Congo hemorrhagic fever virus. †Retired, civil servant, factory, worker, or housewife.

**Table 2:** Multivariable logistic regression of risk factors for infection with CCHFV, Turkey, January–April 2009*

Characteristic	Odds ratio (95% CI)
Age, y	
18–29	1
30–39	0.965 (0.630–1.480)
40–49	1.034 (0.669–1.599)
50–59	1.297 (0.832–2.023)
60–69	2.687 (1.723–4.191)
≥70	4.176 (2.638–6.611)
Education	
None	1
Elementary school	0.977 (0.736–1.297)
High school or university	0.580 (0.357–0.942)
Occupation	
Unemployed and other†	1
Farming	1.688 (1.301–2.190)
Animal husbandry	1.299 (0.922–1.832)
History of tick bite	2.292 (1.768–2.971)

*CCHFV, Crimean-Congo hemorrhagic fever virus. †Retired, civil servant, factory worker, or housewife.

None of the study population had been given a diagnosis of CCHF or had been hospitalized for an acute febrile illness or severe bleeding compatible with CCHF. During 2002–2009, a total of 1,806 adults were given a diagnosis of CCHF in the outbreak region where we conducted our study (Ministry of Health, Turkey). Direct standardization of age-specific seroprevalence rates for the study population with ages of persons in the entire region showed that 15,156 infections would be expected during 2002–2008 (observed:expected ratio 0.12; 95% CI 0.114–0.125). This finding shows that only 12% of the infections were diagnosed and 88% were subclinical.

## Conclusions

We found that the seroprevalence of CCHF in the study region was 10%. Hoogstraal et al. reported that the expected seroprevalence of CCHF was ≈10% during epidemics (*9*). In a small survey in Turkey, IgG against CCHFV was detected in 12.8% of the population in high-risk areas (*10*). Our study showed that the distribution of seropositivity increased with age (Figure 2). This finding was unexpected and showed that CCHF was present in the region long before it was recognized. However, we could not assess whether incidence or severity were amplified in recent years, which led to its detection in 2002. Seroprevalence surveys with representative samples at regular intervals may be the only way for determining if incidence of infection has increased.

Another useful result from this study was the ability to predict that ≈90% of CCHFV infections were subclinical (Technical Appendix). This finding has clinical and epidemiologic implications. First, it shows that the spectrum of severity is highly skewed toward milder disease, although CCHF is believed to be a severe infection similar to other hemorrhagic fevers such as those caused by Ebola or Marburg viruses. Epidemiologically, information about subclinical cases is necessary for estimating the level of herd immunity in the population and predicting the characteristics of the outbreak. Finally, we do not know precisely why CCHF develops into a serious or fatal disease in some patients but is only a mild or asymptomatic in others (*11*). Some studies have shown that factors such as immune response of the host, viral load, or lack of some receptors may affect the clinical form of infection (*12**,**13*). Some authors have suggested that geographic variation in pathogenicity of the virus (*14*) may also be a factor in severity, although the supporting data are lacking (*15*). Therefore, timely detection and comparison of different clinical groups will be helpful in understanding the pathogenicity of the virus or host responses and developing effective treatments for infection.

## Supplementary Material

Technical Appendix Age group–specific seroprevalence rates for infection with CCHFV in the study population, Turkey, January–April 2009.
