# Supplementary material for: Subclinical Infections with Crimean-Congo Hemorrhagic Fever Virus, Turkey
Source: Emerg Infect Dis. 2012 Apr;18(4):640–2. doi: 10.3201/eid1804.111374 (PMC3309668; doi:10.3201/eid1804.111374)
Supplement: Technical Appendix — Age group–specific seroprevalence rates for infection with CCHFV in the study population, Turkey, January–April 2009. [file 11-1374-Techapp_1p.pdf]

Article DOI: http://dx.doi.org/10.3201/eid1804.111374

Subclinical Infections with Crimean-Congo Hemorrhagic Fever Virus, Turkey

Technical Appendix

Technical Appendix Table. Age group–specific seroprevalence rates for infection with CCHFV in the study population, Turkey, January–April 2009*

Age, y	No. persons at risk	Person-years at risk during 2002–2009	No persons positive for CCHFV†	Expected no. infections
18–29	258,844	1,907,420	2.308	4,779
30–39	167,738	1,281,808	2.059	2,763
40–49	143,897	994,722	1.844	2,123
50–59	116,650	872,738	1.849	1,725
60–69	85,998	787,057	2.656	1,828
≥70	78,334	632,846	3.095	1,939
Total	851,461	6,476,590	2.262	15,156

*Changes in the population caused by deaths and migration have been adjusted according to numbers obtained from Turkish Statistical Institute. CCHFV, Crimean-Congo hemorrhagic fever virus.
†Seropositivity per 1,000 persons/year.
